# Professional Advancement

**Published:** 1861-06

**Authors:** S. Clippinger


### PROFESSIONAL ADVANCEMENT.

BY DR. S. CLIPPENGER.

(Read before the Mad River Valley Dental Association.)

Gentlemen and Brethren:—In accordance with your request at our last meeting, I have concluded to pen a few thoughts on a subject that suggests itself to my mind. When appointed, I refused to comply with your request, because I felt incompetent for the task. Though the heart was willing, yet, doubting the ability of my head, I felt that the duty should devolve on some of those among us more competent to perform it. Not willing, however, to prove recreant to the profession, nor to betray the confidence placed in me by my brethren, and feeling that a bad promise is better broken than kept, I resolved to make the effort. And on gaining this point, the sacrifice of self for the good of the society, the question presented itself, How can I, and each of us, best promote the interests of that profession in which we respectively hold our positions as practitioners? Feeling that it is the duty of each of us to throw in his mite towards sustaining the once degraded, but now honored and respected profession, that we may ere long see it standing untrammeled and undisgraced by quacks and quackery, that all who come within its borders may be stimulated to be men of honor and morality, and acting in accordance with this feeling, we will see the prejudice, now existing in a portion of the community, give place to confidence in our efforts to restore, preserve and protect those parts of the human system allotted to our care.

Then, I ask, how shall we best promote the interests of the profession? On this subject much has been said, and yet we feel there is room for much more; for it is a point that will always need attention. We do not expect to advance any thing new at this time, but merely to sound the alarm again, that we may cast about, on the look-out for breakers ahead, lest, thoughtless of each other and the profession at large, we should lose sight of our noble aspirations, and, instead of aiding our standard-bearers to unfurl the banner of Dental Science, be found plucking laurels from its wreath of fame, to accomplish our own selfish designs. Let us then, brethren, divest ourselves of the love of filthy lucre, that we may go to the world holding up each other's hands, and, with unselfish hearts presenting our cause in such manner that it may be seen and felt that we stand united in this glorious cause, and having grounded our weapons of warfare, that our purposes are one; that we all aim at the elevation and promotion of our profession, whose object is the preservation and restoration of the beautiful features of the human family. Let us seek, anxiously, to avail ourselves of all honorable means to increase our little stock of professional knowledge, that we may be worthy of the confidence which society bestows on us. Let us also prove ourselves to be men of integrity, and we shall, ere long, enlighten that portion of society that has been duped by the would-be dentists, who have persuaded them that their quackery is cheap, because they operate for half the price that we do. And let us leave these tinkers to their own ways, showing to the world that they are not our competitors, and that we are not responsible for their malpractices. Again, I say, let us hold up each others' hands, forming an unbroken phalanx, that at last we may be victorious, not only over these our foes, but each one of us over jealous and selfish self.

